# A new Eocene anagalid (Mammalia: Euarchontoglires) from Mongolia and its implications for the group’s phylogeny and dispersal

**DOI:** 10.1038/s41598-018-32086-x

**Published:** 2018-09-17

**Authors:** Sergi López-Torres, Łucja Fostowicz-Frelik

**Affiliations:** 1 Department of Evolutionary Paleobiology, Institute of Paleobiology, Polish Academy of Sciences, Warsaw, PL-00-818 Poland; 2 Key Laboratory of Vertebrate Evolution and Human Origins, Institute of Vertebrate Paleontology and Paleoanthropology, Chinese Academy of Sciences, Beijing, 100044 People’s Republic of China; 3 Center for Excellence in Life and Paleoenvironment, Chinese Academy of Sciences, Beijing, 100044 People’s Republic of China

## Abstract

Anagalidae are extinct primitive Euarchontoglires from Asia, regarded as relatively closely related to basal Glires. So far, the group has been reported only from China and stratigraphically spans from the early Paleocene to the latest Eocene/earliest Oligocene. Anagalids are characterized by a relatively full dental formula featuring slightly enlarged semi-procumbent incisors, prominent canines, and tall cheek teeth with usually heavily worn crowns, indicative of an abrasive diet. Here we report a new genus and species from the late Eocene Ergilin Dzo Formation in southern Mongolia. The first non-Chinese anagalid is also the northernmost record of the family. *Zofiagale ergilinensis* gen. and sp.nov. is remarkable for its relatively small size (comparable only to the Paleocene genera *Huaiyangale* and *Stenanagale*), lack of P_1_, and molariform teeth showing almost no wear, suggesting a different diet than most Anagalidae. Furthermore, its molars display a strong buccal cingulum, a character in anagalids shared only with *Wanogale*. Our phylogenetic analysis of representatives of all anagalid genera based on 82 dental characters places *Anagale* and *Anaptogale* as the most basal lineages and clusters *Zofiagale* gen. nov. together with *Qipania* and *Hsiuannania*. These results suggest three independent northward dispersal events within the family in the late Eocene.

## Introduction

Anagalidae is an enigmatic and poorly studied group of primitive members of Euarchontoglires^1–3^ known from the Paleogene of Asia, with its geographic distribution so far restricted to China^4,5^. The only genus ever considered to be an anagalid from outside of China, namely *Khashanagale* from the Gashato Formation in Mongolia^6^, was reassigned later by McKenna and Bell^7^ to Oxyclaenidae, a condylarth-related group of poorly recognized relationships.

The taxonomic status of anagalid genera is contested and different sources consider true anagalids a different number of genera. The first and only phylogenetic analysis of anagalids was done by Hu^4^, who considered just seven of the 14 genera to be unquestionable anagalids^4,8–14^. The seven genera were *Anagale*, *Anagalopsis*, *Linnania*, *Huaiyangale*, *Hsiuannania*, *Eosigale*, and *Qipania*. Hu^4^ classified *Stenanagale*, *Anaptogale*, and *Diacronus* tentatively within Anagalidae, but excluded *Khashanagale*, *Chianshania*, and *Wanogale* from the family.

In a recent work, Li^5^ considered the three genera that Hu^4^ removed from the family as tentative anagalids, and also included *Interogale* (described by Huang and Zheng^15^) within? Angalidae, a genus that Hu^4^ did not discuss.

Anagalids were never common, but their fossil record drops substantially after the middle Paleocene (Nongshanian Asian Land Mammal Age), with the only presence of *Anagale* from the late Eocene (Ulangochuian ALMA) and *Anagalopsis*, estimated generally in a broader frame of the earliest Oligocene^9^, (but see Zhang and Wang^16^ for an earlier estimate). No anagalids have been reported from the early Eocene (Bumbanian and early Arshatan ALMAs) and most of the middle Eocene (recently Li *et al*.^17^ described unidentified anagalid remains from Irdinmahan deposits of Wulanhuxia in Nei Mongol). Because of the aforementioned uncertain date of Shanmacheng (=Shih-ehr-ma-ch’eng as reported in Bohlin^9^), the type locality of *Anagalopsis kansuensis*, it can be hard to determine the more precise date of extinction of this group of early Euarchontoglires. However, anagalids were certainly present in Asia for nearly 30 million years.

**Figure 1:**
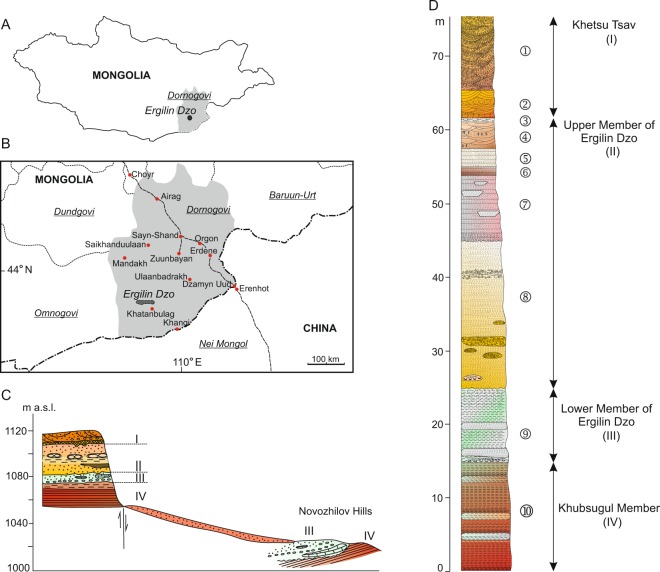
Geography and stratigraphy of new anagalid finding. Map of Mongolia, with the Dornogovi Province (light gray) and the location of Ergilin Dzo (**A**). Detailed map of the Dornogovi Province with the position of the Ergilin Dzo region (**B**). Generalized cross section of late Eocene deposits in the Ergil Obo locality and the Novozhilov Hills; SE part of the Ergilin Dzo Promontory (**C**). Stratigraphic reference section of the Ergilin Dzo Fm. at Ergil Obo, indicating the main beds (I–IV) and sediment layers (1–10; see Supplementary Information for details) (**D**). Modified from Yanovskaya *et al*. 1977 (**C**,**D**). (The drawings were created in Corel Draw X4 (v. 14.0) by Łucja Fostowicz-Frelik).

Here we describe a new anagalid from the late Eocene of the Ergilin Dzo Formation in Mongolia (Fig. 1), the first member of Anagalidae found outside of China, and the northernmost representative of the group. It is characterized by a unique dental morphology and relatively small size. We assign this specimen to a new genus and species. Furthermore, we aim at reconstructing the phylogenetic relationships within Anagalidae in only the second cladistic analysis after Hu’s^4^, using an extended character-data matrix.

## Results

### Systematic Paleontology

Superorder **EUARCHONTOGLIRES** Murphy *et al*. 2001

Order **ANAGALIDA** Szalay & McKenna, 1971

Family **ANAGALIDAE** Simpson, 1931

Genus ***Zofiagale*** gen. n.

urn:lsid:zoobank.org:act:51CCC038-A454-4217-B849-6242FEE6F84C

Type species: *Zofiagale ergilinensis* sp. n., monotypic.

Etymology: Named after Professor Zofia Kielan-Jaworowska (1925–2015), for her exceptional contributions to the understanding of early mammalian fauna from Mongolia; and *-gale*, γαλῆ (feminine), Greek, meaning “weasel”, a common suffix used for the names of anagalids.

Distribution: As for the type and only species.

Diagnosis: As for the type and only species.

**Figure 2:**
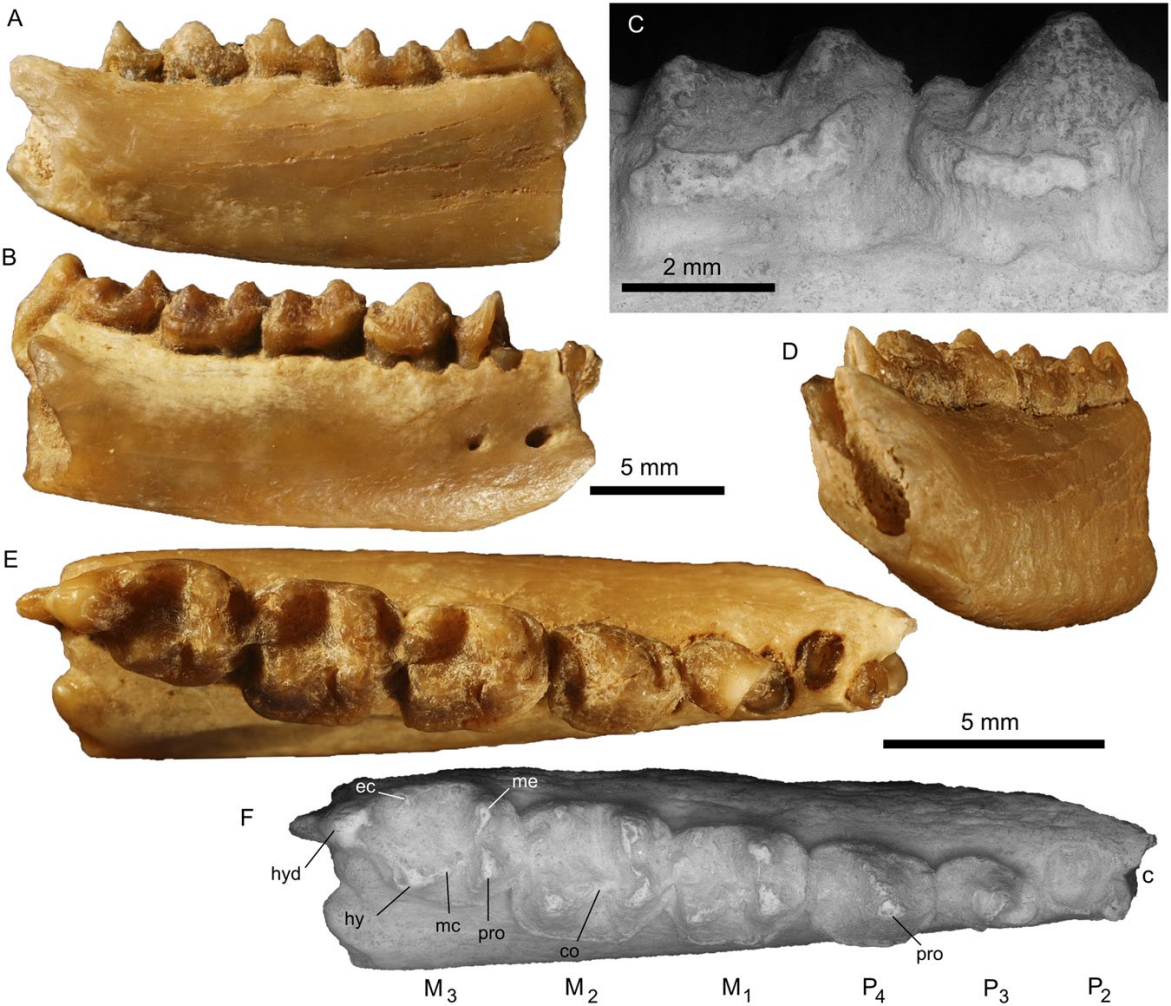
Left mandible body of *Zofiagale ergilinensis* gen. et sp. nov. The holotype and only specimen (ZPAL MgM-II/100) with complete P_4_–M_3_, fragment of P_3_, and roots of P_2_ (**A**–**F**). Lingual (**A**), buccal (**B**,**C**), anterolingual (**D**), and occlusal (**E**,**F**) views, respectively. Magnification of the buccal sides of P_4_–M_1_ showing well-developed cingula (**C**). Note depth and inclined course of alveolus for semi-procumbent canine (**D**). Explanatory SEM image for dental loci and morphology (**F**). Abbreviations: c, canine; co, cristid obliqua; ec, ectoconid; hy, hypoconid; hyd, hypoconulid; mc, mesoconid; me, metaconid; pro, protoconid. (Figure created in Photoshop CS5 and Corel Draw X4 (v. 14.0) by Łucja Fostowicz-Frelik).

*Zofiagale ergilinensis* sp. n.

urn:lsid:zoobank.org:act:1CB04445-F10A-40F5-809A-A6F4EE741C95 Fig. 2.

Holotype: ZPAL MgM-II/100 fragmentary right dentary with the canine alveolus, P_2_ roots, and P_3_–M_3_ *in situ*; housed at the Roman Kozłowski Institute of Paleobiology, Polish Academy of Sciences, Warsaw, Poland (ZPAL).

Type locality and age: Ergilin Dzo locality, Ergilin Dzo Formation, Dornogovi Province, Mongolia (Fig. 1); Ergilian ALMA, late Eocene.

Diagnosis: Smaller than *Anagale*, *Anagalopsis*, *Hsiuannania*, and *Qipania*; comparable size to *Linnania*, *Huaiyangale*, *Eosigale*, and *Interogale*; and larger than *Stenanagale*; based on area of M_1_. Further differs from *Anagalopsis*, *Qipania*, and *Interogale* in having a smaller canine alveolus. Further differs from all anagalids, except for *Interogale*, in lacking a P_1_. Differs from *Anagale*, *Anagalopsis*, and *Linnania* in lacking a diastema between P_2_ and P_3_. Further differs from *Linnania* and *Eosigale* in lacking a diastema between P_3_ and P_4_. Moreover, it differs from all anagalids, except for *Eosigale*, in lacking a metaconid on P_4_ and further differs from *Anagale* and *Linnania* in lacking a paraconid on P_4_. From *Qipania* and *Interogale* it differs in lacking a cristid obliqua on P_4_. Further differs from *Linnania lofoensis* in lacking a paraconid on M_1_, and differs from *L. lofoensis*, *Interogale*, and *Wanogale* in lacking a paraconid on M_2_. Further differs from all anagalids, except for *L. lofoensis*, *L. qinglingensis*, *Interogale*, and *Wanogale* in having a hypoconulid on M_1_–M_2_. Further differs from *L. lofoensis* and *Interogale* in lacking a paraconid on M_3_, and differs from all anagalids, except for *Wanogale*, in having a buccal cingulum on the lower molars. Additionally it differs from *Anagalopsis*, *Linnania*, *Hsuiannania*, and *Qipania* in having the enamel not extending into the alveolus.

Description and comparisons: The right body of the mandible is broken at the level of the canine alveolus in the rostral end, and at the level of the M_3_ talonid in the caudal end, exposing the posterior root of the M_3_. Two mental foramina are visible. The anterior mental foramen is located below the posterior root of the P_2_, and the posterior mental foramen is smaller and located below the posterior root of P_3_. The total length of the P_4_–M_3_ tooth row is 14.5 mm (the total molar row length is 11.5 mm; see Table 1 for more detailed dental measurements). The buccal height of the body of the mandible at the P_4_/M_1_ inter-alveolar septum is 5.7 mm, while at the lingual side it is 6.1 mm.

**Table 1:** Dental measurements of *Zofiagale ergilinensis* gen. and sp. nov. (in mm). Abbreviations: *nd* = no data.

Measurement	Locus				
	P_3_	P_4_	M_1_	M_2_	M_3_
Total length	*nd*	3.02	3.38	3.41	4.69
Trigonid width	*nd*	2.56	2.75	3.25	2.88
Talonid width	1.47	2.14	2.74	3.28	2.69
Trigonid buccal height	*nd*	2.68	2.64	2.62	2.39
Trigonid lingual height	*nd*	2.48	1.88	2.17	1.94
Talonid buccal height	1.51	2.01	2.40	2.32	2.69
Talonid lingual height	1.30	1.76	1.42	1.62	2.09

The lower dental formula for *Zofiagale ergilinensis* is inferred to be? 0.1.3.3. *Zofiagale* is characterized by the loss of P_1_, which is rare in anagalids. Indeed, only *Interogale*, which has been tentatively classified as an anagalid in Li^5^ is known to lack a P_1_ as well^15^. Another rare characteristic for an anagalid seen in *Zofiagale* is the presence of a buccal cingulid on M_1_–M_3_, whereas most anagalids have a completely smooth buccal surface. There is only one other instance of an anagalid with a buccal cingulid: *Wanogale*^12^. *Wanogale* is known from only an M_2_, and also has another feature more typical of euarchontans, which is the presence of a precingulid.

The dentition anterior to P_3_ is lost, and only two roots and a large alveolus remain. The large alveolus is here considered to have hosted a moderately large, semiprocumbent canine. All the other anagalids for which information about the canines is known (*Anagale*, *Anagalopsis*, *Eosigale*, *Qipania*, and *Interogale*) show that they had relatively larger and stronger canines than those of, e.g., zalambdalestids, a group of Cretaceous Eutheria proposed as primitive members of Anagalida *sensu lato*^6^. The semiprocumbent canines are known for *Anagale* and *Qipania*. No anagalid has a P_1_ that is significantly larger than the rest of premolars, therefore it is more likely that the large alveolus hosted a canine rather than a P_1_. The two roots between the canine and the P_3_ could be interpreted either as a double-rooted P_2_ with loss of P_1_, or as a single-rooted P_1_ and a single-rooted P_2_. We favor the first interpretation, even taking into account that the loss of P_1_ is rare in anagalids, and only *Interogale* seems to have lost this tooth position, as some morphological traits strongly imply a two-rooted condition on P_2_. The first of the two roots is shifted buccally with respect to the second root, a pattern that coincides with the root positions in P_3_ (indeed, the anterior root of P_3_ is displaced more buccally than the posterior root, see Fig. 2E,F). This pattern is also seen in other mammals in which there is crowding of the premolars and no lower diastema, such as in the scandentian *Ptilocercus lowii* (e.g., USNM 488054). Therefore, we interpret the buccal torsion of the P_3_ and the lack of diastemata as a clue that the two roots belong to one double-rooted P_2_, the P_1_ has been lost, and that there is a fairly high degree of crowding of the premolars in *Zofiagale*.

The P_3_ is broken, with most of the trigonid missing, but the two roots are preserved. Because of the crowding of the anterior dentition, there is no diastema distal to P_3_ in *Zofiagale*, whereas it is observed in *Linnania*, *Eosigale*, and *Stenanagale*. The remains of P_3_ indicate presence of a large, tall and presumably single-cusped (protoconid?) trigonid and a small saddle-like talonid (Fig. 2A,B,E,F). The talonid is not basined and its buccal and lingual margins are much lower than the distal one, indicating the presence of a minuscule hypoconulid. Also, the lingual margin of the talonid bears a well pronounced cingulid-like edge.

The P_4_ has no metaconid, usually present in other anagalids. The only other anagalid known to lack a metaconid is *Eosigale*. The paraconid is also absent, although a paracristid is clearly visible. The lack of paraconid is more common in anagalids, and only *Anagale* and *Linnania* retain one. The protocristid directed linguodistally is strong and slopes lingually to the lingual side of the tooth, in fact somehow replacing functionally the missing metaconid (see Fig. 2F). The talonid is not basined and is similarly saddle-like as in P_3_, but the distal margin bears a more pronounced eminence, which is most probably a nascent hypoconulid, developed fully in molars.

The mesiodistal length of P_4_ is smaller compared to that of M_1_. This feature is shared by most anagalids, except for *Eosigale* and *Qipania*, which have similar mesiodistal lengths of the two tooth positions. The P_4_ does not have a cristid obliqua, which is rarely present in angalids (only found in *Qipania* and *Interogale*). The P_4_ has a buccal cingulid, not seen in any other anagalid.

The M_1_ does not have a paraconid, although the paracristid is present, and the trigonid basin is deep (Fig. 2F). Many characters of the dental crown, such as small cusps are sometimes difficult to observe in anagalids, as the crowns in most species are worn down very quickly in ontogeny. In any case, the presence of paraconid on M_1_ has only been found in *Linnania*. The trigonid and the talonid of M_1_ have approximately the same area, and the trigonid is less than double of the height of the talonid. The metaconid is higher than the protoconid, and the hypoconid and the entoconid have approximately the same height. The talonid is deeply basined, but the basin area is not very extended. There is a distinct cristid obliqua and small mesoconid at M_1_. The entoconid is higher than hypoconid but its area is slightly smaller than the latter; it is also placed slightly more mesially than the hypoconid. A cusp-like, rounded hypoconulid is placed centrally at the distal margin of the tooth. The tooth bears a strong buccal cingulid, which causes some extension of the buccal side of the tooth.

The M_2_ is larger than M_1_ (Table 1) and more extended buccally. It also does not have a paraconid, but the paracristid is strong and the trigonid basin larger. This contrasts with the expression of the paraconid in *Linnania*, *Interogale*, and *Wanogale* (although there are generic differences in that respect). The metaconid is higher than the protoconid, and the hypoconid and the entoconid are approximately the same height. Both M_1_ and M_2_ have similarly developed hypoconulids, only present in a few other anagalids (*Linnania*, *Interogale*, and *Wanogale*). The talonid basin has a greater area than in M_1_. A weak metaconid and cristid obliqua are present.

The M_3_ is smaller than M_2_ and also has no paraconid, although the paracristid is present. This contrasts with *Linnania* and *Interogale*, which have a paraconid in this locus. The trigonid and talonid are similar in breadth. This is a rare feature in anagalids, since all other anagalids, except for *Anagalopsis*, have narrower talonids than trigonids. The talonid is taller than the trigonid, which is due to the upward curvature of the M_3_ around the area of the hypoconulid lobe. This feature also occurs in *Huaiyangale*, *Eosigale*, and *Qipania*. The hypoconid and the entoconid are approximately the same height, but they are not quite aligned, the entoconid being marginally more distal. The talonid basin is extended and shallower having a relatively flat bottom; it forms a small extension towards a large and distally elongated hypoconulid and invades its surface to some extent (Fig. 2F).

All molars have a distinct hypoflexid. The enamel does not extend into the alveolus, in contrast to a few anagalids (*Anagalopsis*, *Linnania*, *Hsiuannania*, and *Qipania*; Hu^4^); furthermore, the molar crowns are relatively wear resistant, a rare trait for anagalids.

### Phylogenetic analysis

In order to assess the phylogenetic relationships of *Zofiagale* with other anagalid genera, we conducted a cladistic analysis. A list of anagalid-specific characters was created based on character diagnoses from Chow *et al*.^10^, Xu^11,12^, Ting and Tong^13^, Ting and Zhang^14^, and Li^5^, in addition to some of Hu’s^4^ characters from his original phylogenetic analysis of the family. These characters have been complemented by characters taken from phylogenies of other groups of Euarchontoglires^1,18,19^ (see Supplementary Material, Table S1). The primitive eutherian *Zalambdalestes lechei* was chosen as the outgroup for Anagalidae following some broader analyses on Glires^1,2,20,21^.

**Figure 3:**
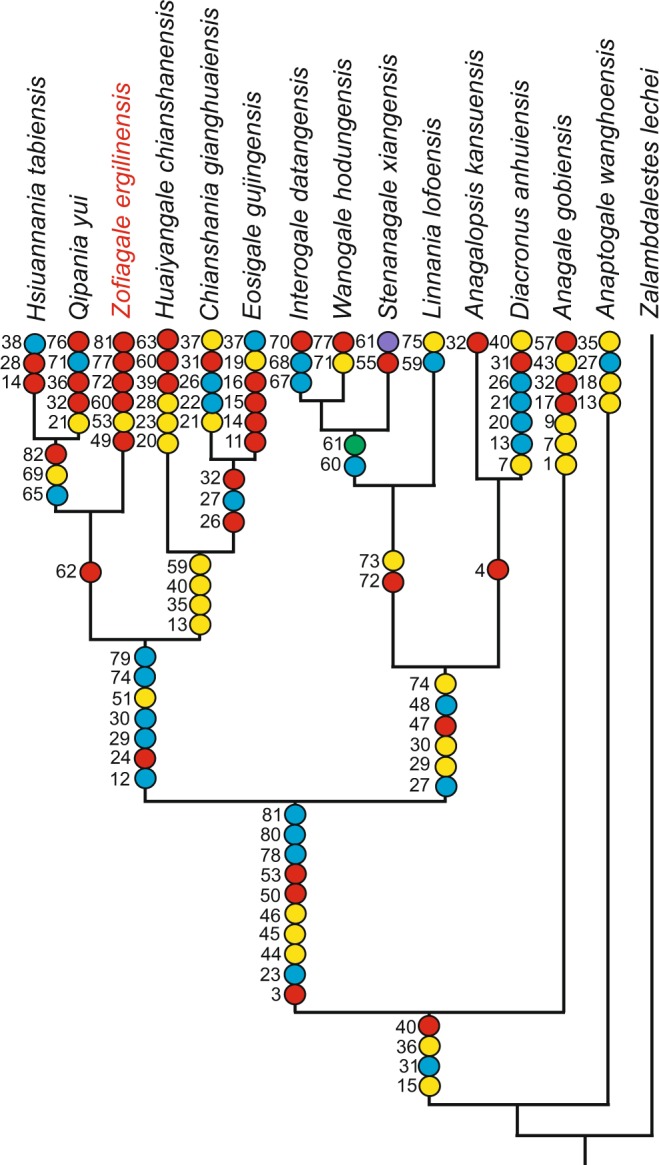
Hypothesis of relationship of known anagalid genera, with listed synapomorphies. The figured tree is the most parsimonious and only resulting tree of the analysis. For further details of the phylogenetic analysis see text and Supplementary Information (Table S1, and Dataset S1). Color code for character states: yellow (0), red (1), blue (2), green (3), and purple (4). (Figure created by Sergi López-Torres with Corel Draw X4 (v. 14.0)).

To correctly place *Zofiagale* in a phylogenetic context, a representative for each of the 14 anagalid genera were included. A total of 82 dental characters were scored for 15 taxa (see Supplementary Information 1). The analysis yielded a single most parsimonious tree (Fig. 3). *Anaptogale* and *Anagale* are the most basal anagalids in this tree, and constitute two separate primitive lineages (Fig. 3). The rest of anagalid taxa are evenly distributed between two distict clades. *Zofiagale* is a sister taxon to *Hsiuannania* and *Qipania*, and, in turn, this clade is the sister group to a clade composed of *Chianshania* and *Eosigale*, and *Huaiyangale* at a more basal position. The second major clade contains a clade grouping most genera presumed tentative anagalids (as per Hu^4^), such as *Stenanagale*, *Wanogale*, and *Interogale*, with *Linnania* being the most basally nested, and *Anagalopsis* clustered with *Diacronus*.

## Discussion

Anagalidae are a poorly known group of placental mammals with no extant representatives. The type and best known genus is *Anagale gobiensis*. By virtue of its almost complete skull and some postcranial remains^8^, *Anagale* has been often used in phylogenetic analyses of Eutheria where it has appeared well nested within Euarchontoglires^1,2,22^. Our results challenge the general topology of Hu’s^4^ hypothesis of relationships among anagalids, relegating *Anagale* to a basal position on our tree, and separating *Linnania* and *Anagalopsis* from *Eosigale*, *Huaiyangale*, and *Hsiuannania*–*Qipania* cluster. Our tree, however, agrees with Hu’s^4^ on the close relationship between *Hsiunnania* and *Qipania*, and *Huaiyangale* and *Eosigale*. Our tree shows a split between two major groups of anagalids. One of these clades includes *Hsiuannania*, *Qipania*, *Zofiagale*, *Huaiyangale*, *Chianshania*, and *Eosigale*. This clade appears well supported by a few synapomorphies: a P^3^ smaller than P^4^, presence of a paracingulum on M^1^, absence of a paraconule and a metaconule on M^1-2^, absence of a diastema distal to P_2_, a talonid taller than the trigonid on M_3_, and a smaller area of M_1_ compared to M_2_. The clade includes four out of seven taxa of Hu’s^4^ unambiguous anagalids. The other major group of anagalids is composed of *Interogale*, *Wanogale*, *Stenanagale*, *Linnania*, *Anagalopsis*, and *Diacronus*. This clade is well supported by the following synapomorphies: presence of a minute parastyle on M^2^, a distinctive paraconule and metaconule on M^1–2^, an erect rather than semi-procumbent lower canine, a large root of the lower canine, and a trigonid taller than the talonid on M_3_.

A new genus *Zofiagale* is well nested within the anagalid tree; in fact, it represents one of the most derived taxa, closely related to *Hsiunnania* and *Qipania*. It shows some unique dental characters which stress its independent phylogenetic heritage and imply a long evolutionary history. Interestingly, *Zofiagale* shares its two most characteristic features with species which do not show the most typical anagalid dental morphology and whose anagalid status has been questioned. With *Wanogale* it shares a strong buccal cingulum on lower molars and crowns that do not heavily wear down, and with *Interogale*, a lack of P_1_. Although Hu^4^ did not consider *Wanogale* to be an anagalid, Li^5^ placed it as a tentative member of the family. On the other hand, *Interogale*, also of questionable status, was considered as belonging to Tillodontia by Wang and Jin^23^.

The taxon sample for our tree does not allow us to decide on what is and what is not an anagalid. In order to do so, other non-anagalid euarchontogliran representatives (i.e., primates, scandentians, lagomorphs, rodents, etc.) should be included in a broader phylogenetic analysis; of course, this raises the question if anagalids are paraphyletic, especially because many tentative anagalids according to Hu^4^ and Li^5^ are well nested within our sample. Such phylogenetic analysis including representatives of euarchontan and Glires lineages is necessary also to properly assess the position of anagalids within the broader framework of Euarchontoglires. However, an analysis of this magnitude is beyond the scope of this study.

**Figure 4:**
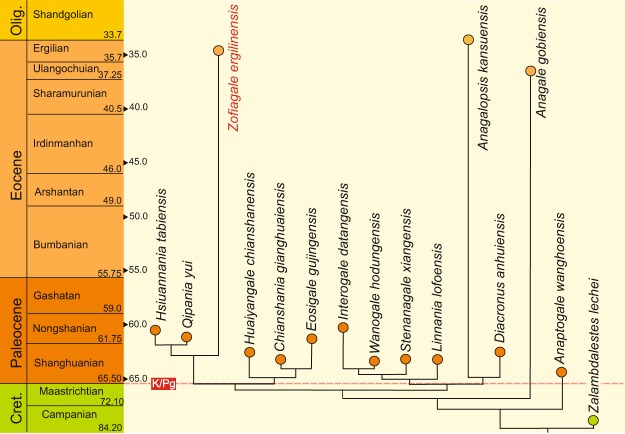
Phylogeny of anagalids with a temporal context. Tree from Fig. 3 plotted against Asian Land Mammal Ages (ALMA). Abbreviations: Olig. = Oligocene; K/Pg = Cretaceous-Paleogene boundary. (Figure created by Sergi López-Torres with Corel Draw X4 (v. 14.0)).

The time bracket for the anagalid evolution spans from the early Paleocene to late Eocene/earliest Oligocene, with the timing of *Anagalopsis* ocurrence still a matter of debate^5,9,16^. Nevertheless, it is one of the last occurring anagalids, together with *Anagale* and *Zofiagale* (Fig. 4). The fact that all three northern anagalids belong to three distinct lineages within the family suggests that disperal events between southern and northern China (including the Mongolian Plateau) were probably common sometime between the late Paleocene and late Eocene, and these happened at least three times within Anaglidae.

**Figure 5:**
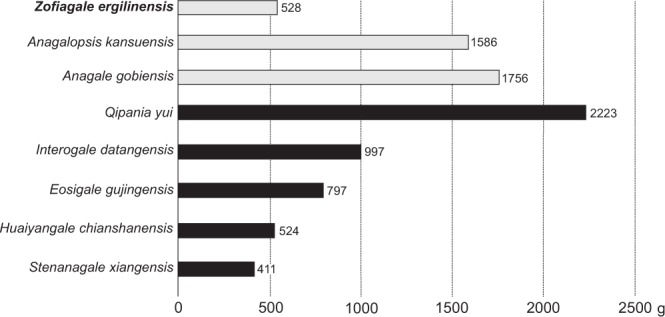
Body mass estimates for representatives of Anagalidae. Based on M_1_ area, following Conroy’s (1987) equation. Light gray, Eocene species; black, Paleocene species. (Drawing made by both authors with Corel Draw X4 (v. 14.0)).

Most anagalids have significantly worn-down cheek teeth, which has led McKenna^24^ to suggest that they may have obtained fairly abrasive food below the surface of the ground. This view endorsed Bohlin’s^9^ idea that anagalids were potentially fossorial. However, back in 1963, the only anagalids known were *Anagale* and *Anagalopsis*, and the variety of dental shape and body masses reported in anagalids have increased greatly (Fig. 5). The present record of anagalids shows taxa with non-worn or lightly worn cheek teeth, such as *Zofiagale* and *Wanogale*. This suggests that their diet could not be primarily composed of abrasive foods, and most probably would differ from that suggested for *Anagale* and *Anagalopsis*. It is, however, worth noting that this inference derives from a sample size of a single specimen and should therefore be taken with caution. Alternatively, ZPAL MgM-II/100 could belong to a very young adult (the M_3_ is erupted) who has not started wearing down the crowns. However, if that was the case and this animal had an abrasive diet, it would be expected to find differential wear between M_1_ and M_3_ (i.e., M_3_ erupts later), and there is not.

*Zofiagale* is one of the smallest anagalids, with an estimated body mass of 516 g, Kay’s^25^ threshold sets a minimum of 500 g for primates to acquire their protein mainly from leaves based on observations of modern primates, with species below that mass being mostly insectivorous. Besides Eocene euprimates, this threshold has been used in Paleocene and Eocene plesiadapiforms and non-primate euarchontans as well^26–29^ and could be tentatively informative in other early Paleogene mammals of generalized morphotypes similar to those of plesiadapiforms. The estimated body mass for *Zofiagale* sits closely to the 500 g threshold, suggesting that an animal this size could easily acquire protein from both insects and leaves, without relying solely on any of the two. Given the blunt cusps, it is likely that the diet of *Zofiagale* might have been largely enriched with fruits. Whereas the presence of a buccal cingulid (rare in anagalids) helps providing gingival protection from the opposing upper tooth and/or food^30–32^, it also effectively broadens the tooth, generating more surface for pressing a bolus of fruit.

## Methods

The specimen was photographed with a scanning microscope Hitachi S−3400 N without coating, in a natural mode (low vacuum) at the Museum and Institute of Zoology, Polish Academy of Sciences, Warsaw, Poland. Furthermore, we documented the specimen using a Keyence digital microscope VHX 5000, and a Nikon (SMZ-800) stereo-microscope at the Institute of Paleobiology (IPAL), Polish Academy of Sciences, Warsaw. The measurements (Table 1) were taken using SYLVAC digital caliper and Keyence digital microscope software with an accuracy to the nearest 0.01 mm. Dental terminology generally follows Meng and Wyss^22^.

### Geological settings

The specimen described here comes from the Ergilin Dzo ridge (“Promontory Bluff”), the locality discovered in 1922 by one of the Central Asiatic Expeditions of the AMNH^33,34^, in the Dornogovi Province, southeastern Mongolia. The specimen was collected in 1963 by a Polish field party during the first Polish-Mongolian Paleontological Expedition^35^. The Polish team prospected only the southeastern part of the ridge of Ergilin Dzo, in the vicinity of Ergil Obo (=Ardyn Obo as reported in Andrews^34^) and Ergil Ula^35^. The Ergilin Dzo ridge is an eminent promontory, ca. 50 km in length, stretching in the EW direction (at ca. 43° 29’ N latitude and 109° E longitude^36^), composed of late Eocene sediment beds, which comprise the Ergilin Dzo Formation^36–38^. The faunal list of fossil vertebrates recovered from Ergilin Dzo currently includes approximately 60 taxa^37^ (see also Supplementary Information), with more than 40 mammals assigned to Eulipotyphla, Lagomorpha, Rodentia, Carnivora, ‘Condylarthra’, Artiodactyla, and Perissodactyla^37,39–45^ (see Supplementary Information for a detailed list and additional references), the last two groups being the most diverse.

The age of the Ergilin Dzo Formation was originally established as pertaining to the Oligocene^36,37^, and the Ergilian ALMA was proposed as an early Oligocene ALMA^36^; it was further correlated with the Chadronian NALMA (North American Land Mammal Age). Because the Chadronian, long considered equivalent to early Oligocene is now placed in the late Eocene as a result of the revised Eocene-Oligocene Boundary^46^, consequently the Ergilin Dzo Fm. also moved into the latest Eocene in age (most probably the middle to late Chadronian; 35.7–33.7 Ma approximate). At present, Ergilin Dzo is a reference section for the Ergilian ALMA, which precedes the earliest Oligocene Shandgolian ALMA^47^. The Ergilin Dzo area during the late Eocene witnessed an increasing aridification^48^ towards forest-steppe, which agrees with paleoenvironmental conditions at the Eocene-Oligocene transition in central Asia^49^.

### Cladistic analysis

A parsimony analysis was performed using TNT 1.5^50^ with all characters equally weighted. Multiple character states were set to be interpreted as polymorphisms, instead of uncertainties. Forty-one of the 82 characters (1, 7, 12–13, 20–23, 26–31, 33–34, 37–38, 40, 43, 46, 48, 53, 57, 59–63, 65–72, 75, 79–82) were ordered based on a morphoclinal criterion, and the rest were left unordered. A traditional search was implemented with 1000 repetitions. The tree-bisection-reconnection (TBR) algorithm was used for branch swapping, with 1000 trees to save per replication.

### Size estimation

We estimated the body mass of *Zofiagale* and comparative anagalid taxa using Conroy’s^51^ equation for prosimians. Although *Zofiagale* is not a primate, Conroy’s^51^ equations have been used to estimate body mass for plesiadapiforms^28,29,51–55^, and anagalids have a more similar generalized morphotype to plesiadapiforms than to modern Glires. Therefore, an equation based on a prosimian subset would be preferable for anagalids, rather than equations based on living rodents^56^ or lagomorphs^57^.

### Nomenclatural acts

This published work and the nomenclatural acts it contains have been registered in ZooBank, the Official Register of for the International Code of Zoological Nomenclature (IZCN), mandatory for electronic-only publications. The ZooBank LSIDs (Life Science Identifiers) can be resolved by appending the LSID to the prefix “http://zoobank.org”. The LSID for this publication is urn:lsid:zoobank.org:pub:65C21FB2-3E88-4A00-8900-6380A4661E81. The identifiers for taxa appear following each new name (see Systematic Paleontology).

## Electronic supplementary material

Supplementary Information

Dataset 1

## Data Availability

All data generated or analyzed during this study are included in this published article (and its Supplementary Information files).
