# Supplementary material for: A new Eocene anagalid (Mammalia: Euarchontoglires) from Mongolia and its implications for the group’s phylogeny and dispersal
Source: Sci Rep. 2018 Sep 17;8:13955. doi: 10.1038/s41598-018-32086-x (PMC6141491; doi:10.1038/s41598-018-32086-x)
Supplement: Supplementary file 1 — Supplementary Information [file 41598_2018_32086_MOESM1_ESM.pdf]

Supplementary Information

A new Eocene anagalid (Mammalia: Euarchontoglires) from Mongolia and its implications for the group's phylogeny and dispersal

Sergi López-Torres[1] and Łucja Fostowicz-Frelik[1,2,3]*

[1]Department of Evolutionary Paleobiology, Institute of Paleobiology, Polish Academy of Sciences, Twarda 51/55, PL 00-818 Warsaw, Poland

[2]Key Laboratory of Vertebrate Evolution and Human Origins, Institute of Vertebrate Paleontology and Paleoanthropology, Chinese Academy of Sciences, Beijing 100044, People's Republic of China

[3]Center for Excellence in Life and Paleoenvironment, Chinese Academy of Sciences, Beijing 100044, People's Republic of China

* Correspondence and requests for materials should be addressed to Ł.F.-F. (email: lfost@twarda.pan.pl)

THE STRATIGRAPHIC SEQUENCE OF ERGILIN DZO

The generalized profile presented in Fig. 1 (main text) was based on a detailed description provided by Yanovskaya et al. (1977) of the Ergil Obo (= Ardyn Obo) section, the most complete columnar section within the Ergilin Dzo (located in the eastern part of the promontory). Additional information on the sediment series of Ergilin Dzo comes from Saneyoshi et al. (2010). Yanovskaya et al. (1977) distinguished four main stratigraphic units, which contain 10 sediment beds in total. These are, from oldest (bottommost) to youngest (topmost): the Khubsugul Member, the upper and lower series of the Ergilin Dzo Member, and the Khetsu Tsav Member (for details, see below). Although Saneyoshi et al. (2010) do recognize the Khubsugul and Khetsu Tsav members, in the lower middle part of the section, they recognize also Sevkhul and Shavag members included by Dashzeveg (1993) below the Ergilin Dzo Member. These two units are better expressed and thicker in other parts of the Ergilin Dzo Formation, Bayan Tsav Obo (BTO) and Shavag (SV); see Saneyoshi et al (2010). Moreover, in contrast to Yanovskaya et al. (1977), Saneyoshi et al. (2010) do not distinguish clearly lacustrine layers within the Ergilin Dzo Member.

Khubsugul Member

[layer 10 of Yanovskaya et al. (1977)] Red mudstones without layering. Clearly marked at the top with red and green. In the lower part, horizontal bed with inter-beds (up to 1 m thick) of green mudstones. According to Saneyoshi et al. (2010) this layer contains a slickenside visible only at the Ergil Obo locality. Also, the presence of a normal fault at the base of the promontory at the Ergil Obo locality was marked by Yanovskaya et al. (1977). Thickness 12–20 m.

Lower Member (lacustrine) of the Ergilin Dzo

[layer 9] Marlstones and white and greenish mudstones layered horizontally with inter-beds of loose light-grey sandstones. Great abundance of the textural trace fossils on the surface of the layers. In the floor of the layer there is a washout underlined with mud-roll conglomerates, with gritstones and small pebbles. Thickness 8–10 m.

Upper Member of the Ergilin Dzo

Contains layers 3–8 of Yanovskaya et al. (1977).

[layer 8] Yellow coarse-grain sands with interbeds of loose mud-roll conglomerates (originated mainly of the crushed and redeposited carbonate nodules and fragments of the underlining lacustrine beds). Saneyoshi et al. (2010) mention fragments of the reworked hardpans (caliches) in the lower part of the Ardyn Obo section in Unit 2 overlaying the Khubsugul Member, probably referring to the observations made by Yanovskaya et al. (1977) for layer 8. In the floor of that layer there is a distinct division marked by a washout between the upper alluvial member and the lower lacustrine one, which serves as a correlation reference point for the strata within the entire Ergilin Dzo Formation. Thickness 18–20 m.

[layer 7] Grey and pink-grey sands, layered and stained with iron oxides, in places solidified into loose sandstones. In the floor of the layer gritstones and in many places visible horizons of washout. Thickness 8–10 m.

[layer 6] Dark brown, clear washed fine-sands, thin-layered horizontally with abundance of iron and manganese oxides.

[layer 5] White, clear washed fine-grain sands, thin-layered horizontally. Thickness 2–3 m.

[layer 4] Pale brown cross-bedded sands, occasionally stained with iron oxide deposits, with lenses of loose sandstones. There is an intensive bioturbation visible in the later and vertically oriented trace fossils (?burrows, 1–6 cm in diameter and 5–30 cm in length; see Yanovskaya et al. (1977) and Saneyoshi et al. (2010), filled with calcite with arenaceous cover. Saneyoshi et al. (2010) refer to such layer being within Unit 3 of the Ergilin Member recognized by them. Thickness 3–4 m.

[layer 3] White, dense sandstones with carbonate content. Thickness 0.5 m.

[layer 2] Yellow to orange-red variously sized cross-bedded sands with intercalations of silts (aleurites) and scarce pebbles. Thickness 2.3 m.

Khetsu Tsav Member

[layer 1] Ochre to brownish gravelstones and pebblestones (shingle) well rounded, in some places solidified into cross-bed conglomerates. The Khetsu Tsav beds are clearly separated from the underlying beds by a distinct but thin border. Thickness of the whole member up to 10 meters.

Comments: Remains of vertebrates were discovered in layers 1 and 7–9. In layer 10 there are no faunal remains (invertebrate or vertebrate). Layers of the Khubsugul Member crop out in the Novozhilov Hills about 2 km NW of Ergil Obo, where in the sandy beds there are fossil vertebrate remains. This layer lays ca. 40 m lower than the Khubsugul layers in the main profile (see Yanovskaya et al. 1977).

LIST OF VERTEBRATE FAUNA OF THE ERGILIN DZO LOCALITY

OSTEICHTHYES

Amiiformes

Amiidae

Amia sp.

Siluriformes

Siluridae

Parasilurus sp.

AMPHIBIA

Anura

Pelobatidae

Uldzinia kurochkini Gubin, 1996

REPTILIA

Testudines

Testudinidae

Ergilemys insolitus (Matthew and Granger, 1923)

Platysternidae

Genus indet.

Emydidae

Genus indet.

? *Melanochelys elongata* Gilmore, 1931

Chrysemys sp.

Trionychidae

Genus indet.

Squamata

Agamidae

Genus indet.

Family indet.

MAMMALIA

Eulipotyphla

Erinaceidae

Palaeoscaptor sp.

Changlelestidae

?*Ictopidium* sp.

Soricidae

Genus indet.

Lagomorpha

Family indet.

Desmatolagus ardynensis Burke, 1941

Rodentia

Cylindrodontidae

Ardynomys olseni Matthew and Granger, 1925

Ardynomys vinogradovi Shevyreva, 1972

Morosomys silentiumys Shevyreva, 1972

Cricetidae

Eumys sp.

Carnivora

Canidae

?*Cynodictis* sp.

Hyaenodontidae

Hyaenodon eminus Matthew et Granger, 1925

Hyaenodon sp. (large form)

Pterodon mongoliensis (Dashzeveg, 1964)

?Condylarthra

Genus indet. (small form)

Perissodactyla

Brontotheriidae

Epimanteoceras robustus (Granger & Gregory, 1943)

Parabrontops gobiensis (Osborn, 1925)

Metatitan relictus Granger & Gregory, 1943

Protembolotherium efremovi Yanovskaya, 1954

Embolotherium andrewsi Osborn, 1929

Embolotherium ergilense Dashzeveg, 1975

Titanodectes ingens? Granger & Gregory, 1943

Chalicotheriidae

Schizotherium avitum Matthew & Granger, 1923

Helaletidae

Colodon inceptus Matthew & Granger, 1925

Colodon sp.

Hyraconodontidae

Ardynia praecox Matthew & Granger, 1923

Genus indet. (small form)

Amynodontidae

Amynodon mongoliensis Osborn, 1936

Cadurcodon ardynensis (Osborn, 1923)

Caenolophus promissus Matthew & Granger, 1925

Gigantamynodon cessator Gromova, 1954

Hypsamynodon progressus Gromova, 1954

Genus indet. (small form)

Rhinocerotidae

Ronzotherium (=*Symphysorrhachis*) *brevirostris* Belyayeva, 1954

Artiodactyla

Entelodontidae

Entelodon (=*Ergilobia*) *gobiensis* (Trofimov, 1952)

cf. *Brachyhyops* sp. (=*Archaeotherium* sp.)

Anthracotheriidae

Brachyodus sp.

Genus indet.

Lophiomerycidae

Lophiomeryx angarae Matthew & Granger, 1925

Lophiomeryx gobiae Matthew & Granger, 1925

Archaeomerycidae

Miomeryx altaicus Matthew & Granger, 1925

Miomeryx sp. (large form)

Gelocidae

Gobiomeryx dubius Trofimov, 1956

Eumeryx sp.

Order indet.

Didymoconidae

Ardynictis furunculus Matthew & Granger 1925

ADDITIONAL REFERENCES

Dashzeveg D. 1991. Hyracodontids and rhinocerotids (Mammalia, Perissodactyla, Rhinocerotoidea) from the Paleogene of Mongolia. Palaeovertebrata 21: 1–84.

Gubin Y.M. 1996. First find of a pelobatid (Anura) from the Paleogene of Mongolia. Palaeontological Journal 30: 571–574.

Młynarski M. 1968. Notes on tortoises (Testudinidae) from the Tertiary of Mongolia. Palaeontologia Polonica 19: 83–97.

Tsubamoto T., Saneyoshi M., Watabe M., Tsogtbaatar K., Mainbayar B. 2011. The entelodontid artiodactyl fauna from the Eocene Ergilin Dzo Formation of Mongolia with comments on *Brachyhyops* and the Khoer Dzan locality. Paleontological Research 15:258–268.

Table S1. Descriptions of dental characters

No.	Upper incisors	
1	Size of I^3 (**ordered**)	0: Larger than I^2; 1: Similar size to I^2; 2: Smaller than I^2
	**Upper canine**	
2	Size of root	0: Small or moderate; 1: Large
3	Height of C^1 relative to P^3	0: Shorter than P^3; 1: Taller than P^3
	**Upper premolars**	
4	Number of P^1 roots	0: 2; 1: 1
5	Size of P^1	0: Moderate; 1: Small
6	Size of P^2	0: Greatly reduced, less than 50% of P^3 width; 1: Smaller than P^3, but larger than 50% of P^3 width
7	Number of P^2 roots (**ordered**)	0: 3; 1: 2; 2: 1
8	Presence of metacone on P^3	0: Absent; 1: Present
9	Presence of protocone on P^3	0: Absent; 1: Present
10	Presence of postparacrista on P^3	0: Absent; 1: Present
11	Presence of parastyle on P^3	0: Absent; 1: Present
12	Size of P^3	0: Larger than P^4; 1: Subequal to P^4; 2: Smaller than P^4
13	Expression of P^4 metacone	0: Absent; 1: Visibly smaller than paracone; 2: As large as paracone
14	Presence of postcingulum on P^4	0: Absent; 1: Present
15	Presence of parastyle on P^4	0: Absent; 1: Present
16	Presence of stylar shelf on P^4	0: Absent; 1: Present
17	Presence of preparacrista on P^4	0: Absent; 1: Present
18	Presence of postparacrista on P^4	0: Absent; 1: Present
19	Morphology of P^4 parastylar lobe	0: Large, projecting; 1: Small, not projecting
20	Buccal/Lingual P^4 crown height proportion (**ordered**)	0: $x < 0.6$; 1: $0.6 < x < 1.5$; 2: $x > 1.5$
21	Molarization of P^4 (**ordered**)	0: Not molarized; 1: Semi-molarized; 2: Molarized
	**Upper molars**	

22	Presence of parastyle on M^1 (**ordered**)	0: Present, well-developed; 1: Minute; 2: Absent
23	Presence of metastyle on M^1 (**ordered**)	0: Present, well-developed; 1: Minute; 2: Absent
24	Presence of paracingulum on M^1	0: Absent; 1: Present
25	Presence of metacingulum on M^1	0: Absent; 1: Present
26	Buccal/Lingual M^1 crown height proportion (**ordered**)	0: $x < 0.85$; 1: $0.85 < x < 0.95$; 2: $x > 0.95$
27	Presence of parastyle on M^2 (**ordered**)	0: Present, well-developed; 1: Minute; 2: Absent
28	Presence of metastyle on M^2 (**ordered**)	0: Present, well-developed; 1: Minute; 2: Absent
29	Presence of paraconule on M^{1-2} (**ordered**)	0: Distinctive; 1: Weak; 2: Absent
30	Presence of metaconule on M^{1-2} (**ordered**)	0: Distinctive; 1: Weak; 2: Absent
31	Morphology of stylar region on M^{1-2} (**ordered**)	0: Wide stylar shelves; 1: Stylar shelves absent, but stylar cingula distinct; 2: Stylar cingula weak or absent
32	Expression of buccal cingulum on M^{1-2}	0: Present; 1: Absent
33	Presence of paracone on M^3 (**ordered**)	0: Large; 1: Small; 2: Absent
34	Presence of hypoconal shelf on M^3 (**ordered**)	0: Enlarged mesiodistally; 1: Reduced or small indentation in the tooth wall; 2: Absent
35	Presence of preparaconule crista on M^3	0: Absent; 1: Present
36	Presence of parastyle on M^3	0: Present; 1: Absent
37	M^1/M^2 area proportion (**ordered**)	0: $M^1 > M^2$; 1: Subequal; 2: $M^1 < M^2$
38	M^1/M^3 area proportion (**ordered**)	0: $M^1 > M^3$; 1: Subequal; 2: $M^1 < M^3$
39	M^2/M^3 area proportion	0: $M^2 > M^3$; 1: Subequal
40	Regressiveness of upper molar buccal roots	0: Small but not regressive; 1: Regressive, crown mainly supported by large lingual roots
	**Lower incisors**	
41	Size of I_1 (**ordered**)	0: Smaller than subsequent; 1: Comparable to subsequent; 2: Larger than subsequent
42	Orientation of I_1	0: Semiprocumbent; 1: Procumbent-horizontal
43	Diastema distal to incisors (**ordered**)	0: Absent; 1: Small; 2: Large

	**Lower canine**	
44	Canine height relative to P_1	0: $C_1 > P_1$; 1: Subequal
45	Canine height relative to P_2	0: $C_1 > P_2$; 1: Subequal
46	Canine height relative to P_3 (**ordered**)	0: $C_1 > P_3$; 1: Subequal; 2: $C_1 < P_3$
47	Orientation of C_1	0: Semiprocumbent; 1: Erect
48	Size of C_1 root (**ordered**)	0: Small; 1: Moderate; 2: Large
	**Lower premolars**	
49	Presence of P_1	0: Present; 1: Absent
50	Number of P_1 roots	0: 2; 1: 1
51	Diastema distal to P_2	0: Absent; 1: Small
52	Diastema distal to P_3	0: Absent; 1: Small
53	Expression of P_4metaconid (**ordered**)	0: Absent; 1: Smaller than protoconid; 2: As large as protoconid; 3: Taller than protoconid
54	Presence of paraconid on P_4	0: Present; 1: Absent
55	Mesiodistal length of P_4 relative to M_1	0: Smaller than M_1 area; 1: Subequal
56	Presence of cristid obliqua on P_4	0: Absent; 1: Present
	**Lower molars**	
57	Area of M_1(**ordered**)	0: $x < 15$ mm^2; 1: 15 mm$^2 < x < 20$ mm^2; 2: $x > 20$ mm^2
58	Expression of paraconid on M_2	0: Reduced; 1: Absent
59	Trigonid/talonid area proportion on M_1(**ordered**)	0: Trigonid larger than talonid; 1: Subequal; 2: Talonid larger than trigonid
60	Length/width proportion of M_1 (**ordered**)	0: $x < 1.15$; 1: $1.15 < x < 1.4$; 2: $x > 1.4$
61	M_1 trigonid length/width proportion (**ordered**)	0: $x < 0.65$; 1: $0.65 < x < 0.75$; 2: $0.75 < x < 0.85$; 3: $0.85 < x < 1$; 4: $x > 1$
62	M_1 talonid length/width proportion (**ordered**)	0: $x < 0.4$; 1: $0.4 < x < 0.6$; 2: $x > 0.6$
63	Relative height of the trigonid compared to the talonid on M_1	0: Trigonid less than double the height of the talonid; 1: Trigonid and talonid of the same height
64	Relative height of the protoconid compared to the metaconid on M_1 (**ordered**)	0: Protoconid higher than metaconid; 1: Subequal; 2: Metaconid higher than protoconid
65	Relative height of the hypoconid compared to the entoconid on M_1 (**ordered**)	0: Hypoconid higher than entoconid; 1: Subequal; 2: Entoconid higher than

		hypoconid
66	Expression of paraconid on M_2	0: Reduced; 1: Absent
67	Length/width proportion of M_2 (**ordered**)	0: x <1.2; 1: 1.2< x <1.5; 2: x > 1.5
68	M_2 trigonid length/width proportion (**ordered**)	0: x <0.5; 1: 0.5< x <0.7; 2: x > 0.7
69	M_2 talonid length/width proportion (**ordered**)	0: x <0.575; 1: 0.575< x <0.7; 2: x > 0.7
70	Relative height of the protoconid compared to the metaconid on M_2 (**ordered**)	0: Protoconid higher than metaconid; 1: Subequal; 2: Metaconid higher than protoconid
71	Relative height of the hypoconid compared to the entoconid on M_2 (**ordered**)	0: Hypoconid higher than entoconid; 1: Subequal; 2: Entoconid higher than hypoconid
72	Presence of hypoconulid on M_{1-2}	0: Absent; 1: Present
73	Presence of paraconid on M_3	0: Present; 1: Absent
74	Relative height of the trigonid compared to the talonid on M_3 (**ordered**)	0: Trigonid less than double the height of the talonid; 1: Trigonid and talonid of the same height; 2: Talonid taller than the trigonid
75	Relative height of the hypoconid compared to the entoconid on M_3 (**ordered**)	0: Hypoconid higher than entoconid; 1: Subequal; 2: Entoconid higher than hypoconid
76	Distinctiveness of molar hypoflexids	0: Distinct, invaginated; 1: Not distinct
77	Presence of buccal cingulid on lower molars	0: Abent; 1: Present
	**Lower tooth row**	
78	Area of P_1 relative to P_2 (**ordered**)	0: $P_1 > P_2$; 1: Subequal; 2: $P_1 < P_2$
79	Area of M_1 relative to M_2 (**ordered**)	0: $M_1 > M_2$; 1: Subequal; 2: $M_1 < M_2$
80	Area of M_1 relative to M_3 (**ordered**)	0: $M_1 > M_3$; 1: Subequal; 2: $M_1 < M_3$
81	Area of M_2 relative to M_3 (**ordered**)	0: $M_2 > M_3$; 1: Subequal; 2: $M_2 < M_3$
	**Upper/lower dentition**	
82	Extension of molar enamel	0: It does not extend into the alveolus; 1: It extends into the alveolus

SUPPLEMENTARY DATA 1. Newick format for trees from Figures 3 and 4

(((((((Hsiuannania,Qipania),Zofiagale),(Huaiyangale,(Eosigale,Chianshania))),((((Interogale, Wanogale),Stenanagale),Linnania),(Anagalopsis,Diacronus))),Anagale),Anaptogale),Zalambdalestes);
